# Biodiversity isn't measured in tons: Making sense of nature in the context of sustainable agriculture

**DOI:** 10.1002/eap.70328

**Published:** 2026-10-10

**Authors:** Iqbal Singh Bhalla, Philip A. Loring

**Affiliations:** ^1^ Department of Geography, Environment and Geomatics University of Guelph Guelph Ontario Canada; ^2^ The Nature Conservancy Arlington Virginia USA; ^3^ Present address: Department of Biology University of Saskatchewan Saskatoon Saskatchewan Canada

**Keywords:** agroecosystems, biodiversity conservation, biodiversity monitoring, food systems transformation, social‐ecological systems, sustainability transitions

## Abstract

In this review, we articulate several important but often overlooked concepts, trends, and perspectives in the biodiversity monitoring space, specifically in the context of transforming agriculture and the wider food system. We describe the common approaches to biodiversity monitoring—those focused on species, communities, functions, habitats, etc.—and the methods and tools commonly used to track change. We then describe a set of fundamental ecological principles that are often overlooked by practitioners entering the agrobiodiversity space for the first time, particularly those with technical, industrial, and policy expertise in agriculture‐adjacent fields who seek to incorporate biodiversity into their research and operations. For these practitioners, we discuss the importance of abundance, the timelines along which population trends can be quantitatively established, the geographical scales at which ecological interactions play out, and the implications therein for anyone looking to monitor the effect of farm management practices. We attempt to describe the complexities that arise when navigating the intersection of ecological, agricultural, and social systems, highlighting the multiple sets of voices, values, and priorities that come into play in this space. From intergovernmental panels to individual farmers, the agrobiodiversity space holds a multitude of arenas where decisions are made. We raise the question of “why, how, and for whom are we monitoring biodiversity?” to encourage practitioners new to this space to consider the implications of their approach. Finally, we discuss the conflicts and trade‐offs that emerge in the intersection of productivity, profits, carbon, and biodiversity, and explore the challenges in finding a balance between them in the transition toward a more sustainable form of agriculture.

## INTRODUCTION

Agriculture covers 40% of the land surface of the earth and produces the food that sustains humanity (Power, 2010). It is also defined by a contradiction. On the one hand, agricultural productivity depends on healthy agroecosystems, which comprise and are sustained by a range of natural processes and biological communities. On the other hand, contemporary global agriculture consumes more natural resources than any other human activity (FAO, 2019) and is one of the most consequential drivers of deforestation (Curtis et al., 2018), environmental pollution (Mekonnen & Hoekstra, 2015), and biodiversity loss (Maxwell et al., 2016; Outhwaite et al., 2022).

For decades, the dominant narrative about food systems has judged farms and farming technologies exclusively by their productivity (Emsley, 2001). As the demand for agricultural productivity and profits has increased, societies have both extensified and intensified agricultural land use (Dornelles et al., 2022). This was more prevalent in developed and industrialized nations, which in the last century have adopted increasingly complex, mechanized, and intensive practices (Loring & Sanyal, 2021), but even countries dominated by smallholder farms have also incorporated elements of intensification, notably the widespread adoption of fast‐growing, high‐input, high‐yield varieties of crops during the green revolution of the 1960s and 1970s (Pingali, 2012). A side effect of this intensification is the damage that has been and continues to be done to the environment (John & Babu, 2021). Synthetic fertilizers brought in to substitute for nutrients in the soil leach into local water bodies. Chemical pesticides and herbicides, sprayed to stem the increasingly resistant pests and weeds (Bottrell & Schoenly, 2012), do great harm to other wildlife and biomagnify up the food chain (Fritsch et al., 2022; Molenaar et al., 2024; Wan et al., 2025). And still, productivity remains the key measure of a farm's quality (Thompson, 2017).

As concepts such as organic, climate‐friendly, and regenerative farming move into mainstream policy (Paolini et al., 2024), and with agriculture under increasing focus for its climate impacts, the discourse is shifting from a singular focus on productivity. Many food systems practitioners and experts now recognize the need for global agriculture to balance long‐term productivity with environmental and societal sustainability (Reganold & Wachter, 2016; Rockström et al., 2017). Though environmental, yield, and social benefits of alternative farming are variable in practice (Seufert & Ramankutty, 2017), and scaling up risks replicating some of the same problems of conventional agriculture (Guthman, 2004), these shifting priorities have nonetheless brought carbon and biodiversity squarely into the conversation in recent years.

With carbon emissions now a mainstay feature of global geopolitics (United Nations, 1992), we are seeing a proliferation of guidelines and regulation around supply chain emissions (European Investment Bank, 2021), followed by a rapid growth of carbon markets and other approaches to carbon financialization. This has led to increased attention on agriculture by policymakers and private industry, who see it as both a source and potential sink of a vast amount of carbon. More recently, nature, often codified as biodiversity, is also being brought up in the context of supply chains and in much the same way (Linsley et al., 2023), and with it a similar shift in who is concerned with agricultural biodiversity. Its monitoring and conservation are no longer the sole purview of government and academia. Private actors have entered this space, and companies increasingly look to farmland as a source of both risk and opportunity (Taskforce on Nature‐related Financial Disclosures [TNFD], 2023).

As we will explain here, however, unlike carbon, biodiversity cannot be measured in tons. Indeed, in the context of measuring or monitoring change over time, and with the aim of assigning value, biodiversity is extremely difficult to define and cumbersome to effectively quantify. There is no nonnormative way to construe biological value (IPBES, 2019). As such, any set of indicators that assign value to biodiversity must be adapted to different ecological and cultural contexts, scales, and time frames, each of which will have unique implications for agricultural productivity, emissions, human and ecosystem health, and justice. As a result, the what, where, and how of biodiversity monitoring remains debated (Herzog et al., 2017; Herzog & Franklin, 2016; Kissling et al., 2026; Proença et al., 2017; Vačkář et al., 2012).

In this review, we synthesize the ecological, methodological, and sociopolitical complexities of characterizing biodiversity in agricultural landscapes. This review is primarily intended for practitioners entering the agrobiodiversity space from adjacent fields—including carbon market designers, supply chain sustainability professionals, agri‐food technology developers, remote sensing scientists, and policymakers tasked with integrating biodiversity into climate or agricultural regulation—who may be unfamiliar with the ecological, methodological, and sociopolitical complexities involved. Many of the points we raise will be familiar to ecologists, agroecologists, and conservation biologists, and especially to Indigenous and local communities who have long managed landscapes for biodiversity. Our aim is not to present novel ecological theory, but to synthesize concepts and considerations that are well established in some disciplines yet routinely overlooked or underappreciated when biodiversity enters policy, market, and technology conversations dominated by other expertise.

We start with key ecological dynamics and considerations relevant to understanding biodiversity change in agricultural landscapes. To keep the discussion manageable, we focus specifically on aboveground biodiversity, acknowledging that belowground and marine/aquatic biodiversity are fields unto themselves and thus merit reviews of their own. We then outline the common methodological and technological approaches in this space, discussing the power of the data they produce. Finally, we explore sociological and cultural aspects of farming that intersect with biodiversity, highlighting the complexity of a system with multiple voices, value systems, and priorities.

## APPROACHES TO BIODIVERSITY MONITORING

Monitoring biodiversity is a fundamental challenge in assessing the impacts of agricultural practices on the natural world. Biodiversity itself is an abstract concept that, depending on which framing you adopt, encompasses living organisms, their genetic diversity, and the ecological assemblages and communities in which they reside. People also parse different subsets of biodiversity as being more or less important or desirable.

In contemporary conservation and agricultural policy, for example, contrasts are commonly drawn between wild biodiversity and on‐farm crop and livestock agrobiodiversity. These aspects of biodiversity are treated as separate categories—sometimes for reasons of theoretical focus, and sometimes because of a genuine disagreement about their relative inherent or instrumental value (Heywood, 2013; Jago et al., 2024). Normally, these two aspects of biodiversity are treated as separate; however, Indigenous Peoples and other traditional farming communities have long recognized wild, agricultural, and cultural biodiversity as interdependent and mutually sustaining (Nabhan, 2012). A substantial body of scholarship demonstrates that biological and cultural diversity are not merely correlated but mutually constituting—that the practices, knowledge systems, and relationships through which communities manage landscapes actively produce and maintain biological diversity, just as biological diversity sustains cultural practices and identities (Lepofsky et al., 2021; Maffi, 2001; Nazarea, 2005). Likewise, conservation organizations and global agreements such as the Global Biodiversity Framework (GBF) make important distinctions about the many valid normative rationales to protect biodiversity, including not just individually threatened species, but also the importance of protecting a wide range of ecosystem types, globally significant ecosystems, and culturally valued ecosystems.

Because it is an abstract, multidimensional, and dynamic concept, biodiversity cannot be measured directly. Instead, we must rely on indicators—tangible, measurable variables that serve as proxies for the underlying biological or ecological conditions that we aim to understand and monitor (Bell & Morse, 2012). Indicators, such as species diversity, are made up of one or more unidimensional metrics, in this case, the abundances of species X, Y, and Z. Whichever indicator or set of indicators we choose involves its own set of assumptions, frameworks, or theories that connect the things we can measure to the version or framing of biodiversity we wish to capture (Hinkel, 2011). Additionally, logistical, technological, and financial considerations can play a significant role in the choice of indicator; the choice of what to measure and report is therefore partly a function of what was feasible to collect. Applied ecological research, thus, invariably involves balancing ecological objectives with practical and resource constraints.

No one indicator can capture every aspect of biodiversity, and as we discuss below, each individual indicator we might choose shows a different degree of sensitivity and selectivity to the patterns of change in an ecosystem. Considered individually, any single indicator can therefore present a misleading picture of ecosystem dynamics (Santini et al., 2017). Most biodiversity monitoring frameworks therefore integrate a number of indicators, the choice of which should reflect the motivations behind the measurement. Table 1 highlights the strengths and limitations of metrics commonly used to represent and track changes in biodiversity. These include multispecies and multi‐taxa measures of diversity, single species metrics whose presence, condition, abundance, or activity act as proxies for broader ecosystem health, and structural metrics.

**TABLE 1:** Metrics commonly used to represent and track trends in biodiversity, their applications, and key considerations for their use and interpretation, divided into multispecies and multi‐taxa composite metrics, single species proxies metrics, and structural metrics.

Indicator	Indicator application	Considerations
Multispecies and multi‐taxa composite metrics		
Species richness	A commonly used indicator that represents the relative variety of species in a region that does not account for species abundance or traits. It can and often does correlate with community‐ and ecosystem‐level dynamics, and as such can be used in combination with other indicators to track responses to disturbance and changing habitat quality (Schmitz et al., 2013).	When considered in isolation, species richness presents a reductive measure of biodiversity that cannot speak to ecosystem functioning or nuance details of turnover such as the loss of endemic species upon the arrival of invasives, or the replacement of habitat specialists by generalists (Hillebrand et al., 2018).
Community indicators	Indicators such as species diversity and evenness incorporate the relative abundance of different species (with or without species richness). By describing how balanced a community is (evenness), along with the variety of species within it (diversity), these indicators can correlate with the ability of a community to respond to disturbance, thus speaking to the stability of an ecosystem (Loreau & de Mazancourt, 2013).	These indicators do not directly describe functional or genetic characteristics of the community. Furthermore, their effectiveness at capturing ecosystem health/resilience can depend on the composition and ecological role of the species included in the calculations. As it is easier to monitor some species/taxa over others, diversity and evenness indicators that are limited to specific taxa can miss important ecological dynamics and functions (Hillebrand et al., 2008).
Trait‐based indicators	Indicators such as functional redundancy, diversity, and evenness consider characteristics and traits of species, thereby, theoretically, linking them to ecological functions and phenomena. When appropriately measured and interpreted, these indicators can speak to the stability and resilience of an ecosystem, its ability to respond to disturbance, and to provide ecosystem services (Cadotte et al., 2011; Hoehn et al., 2008; Mouillot et al., 2013).	The use of trait‐based indicators relies on a number of decisions regarding the choice of taxa and scales of measurement, the selection and weighting of traits, and how they are linked to ecosystem function. This can make trait‐based indicators challenging to appropriately contextualize and interpret (Messier et al., 2010; Petchey & Gaston, 2006).
Single species metrics		
Ecosystem service providers	Ecosystem services describe functions of natural and managed systems that provide value to humanity (Costanza et al., 1997; Millennium ecosystem assessment, 2005). Biological communities provide multiple services to us such as filtering water (Malinga et al., 2018), maintaining soil structure and quality (Blouin et al., 2013), regulating pests and pollinating flowers (Zulka & Götzl, 2015). By monitoring the trends in species and taxa providing ecosystem services, one can track the value of these services, and design targeted interventions to bolster their delivery.	Monitoring a species only indirectly estimates the value of the provided service, and has a different degree of accuracy in different contexts/services. In the case of pollination or seed dispersal, the correlation between species activity and ecosystem service delivery might be quite strong. In the case of pest control, other factors may bolster, reduce, or even negate the value provided by a species (Karp & Daily, 2014).
Surrogate/sentinel species	Bioindicator species are those used to track changes in environmental quality, ecological disturbance, or the responses of other species or groups (McGeogh, 1998). Effective indicator species, such as frogs (Babini et al., 2018), earthworms (Falco et al., 2015), insectivorous bats (Jones et al., 2009), and birds (Mansfield et al., 2024), exhibit specific traits which include (1) being common and easy to monitor in the study area, and at a scale appropriate for the project, (2) showing a predictable response to changes in specific environmental features across a wide range of intensities, and/or (3) correlating strongly with responses of other species, taxa, trophic levels or functional groups (McGeogh, 1998; Noss, 1990). Keystone species have an outsized role in shaping ecosystem dynamics either by offsetting competitive advantage among prey species or via the interspecies facilitative effects of ecosystem engineering (Paine, 1995). Flagship species are often large, top‐of‐the‐food‐chain species, and can also be used as indicators of ecosystem characteristics—such as habitat quality or connectivity—but are more often used for their “charismatic appeal” to elicit support for conservation projects (Segura et al., 2014). By acting as a proxy for other features or communities of interest, surrogate species can vastly reduce the effort needed to monitor biodiversity and signal impending ecological change.	Care should be taken to select species whose response to various biological taxa and environmental conditions has been systematically tested (Rodrigues & Brooks, 2007), as misinterpretation of a bioindicator's response can have significant policy implications (Russo et al., 2021). This is particularly important in the case of flagship species, often chosen for their symbolic, commercial, and/or public interest value, which may not reflect their ecological role or value (Andelman & Fagan, 2000).
Cultural keystone species	Plant or animal species that hold significant cultural importance for Indigenous or local communities, often serving cultural, nutritional, medicinal, or spiritual roles (Garibaldi & Turner, 2004). By identifying the species that are most culturally meaningful and economically critical to the people who directly interact with and depend on local ecosystems, cultural keystone species bring in valuable traditional ecological knowledge that can complement Western scientific frameworks (Ijatuyi et al., 2025).	Partnerships with local knowledge holders and elders are necessary to identify and interpret the role of cultural keystone species, specifically regarding what their status, prevalence, and abundance signifies with respect to environmental health and trends of change. Also, that a species matters as a cultural keystone does not necessarily mean that it is a particularly abundant species, but rather that the species occupies a position as an important nexus between biological and cultural systems.
Structural metrics		
Habitat	The extent, condition, connectedness, and other features of a habitat in a landscape can be very informative of the state of the ecosystem as a whole. Habitat structure can speak to the complexity of an ecosystem and relate to the diversity of communities supported and therefore ecological properties of an ecosystem (Godbold et al., 2011), while habitat area, connectivity, and fragmentation levels can relate to ecosystem resilience (Hodgson et al., 2011). Habitat features, in combination with abiotic conditions, can also be used to predict features such as occurrence, distribution, and genetic diversity of species using techniques such as species distribution modeling (Franklin, 2023; Waldock et al., 2022).	Habitat indicators gleaned purely from remote‐sensed methods, while scalable, can miss nuances crucial for a comprehensive ecosystem assessment (Cavender‐Bares et al., 2022). Consider for example an agroforestry system sprayed with pesticides. High scores for habitat structure and connectedness would not reflect what might be a struggling ecosystem bereft of insects and insectivores. The accuracy of species distribution models, meanwhile, can vary greatly, influenced by the quality and interpretation of species occurrence datasets (Jenkins et al., 2003), model transferability from one location to another, and model parameterization and calibration (Lee‐Yaw et al., 2022).

## THE IMPORTANCE OF ABUNDANCE

Population abundance is an important feature of ecosystems (Hillebrand et al., 2018) that is well represented as a metric in agroecological research (Bengtsson et al., 2005), sometimes included in agri‐environmental scheme design (Elmiger et al., 2023; Hagemann et al., 2024; Kleijn & Sutherland, 2003), and frequently overlooked in biodiversity credits and offsets (Marshall et al., 2024, 2020). In the case of ecosystem services, the value provided by a species is often proportional to its population size (or activity levels) (Gaston et al., 2018; Harrison et al., 2014). The same is true of disservices such as crop damage by pests. Merely monitoring the presence of a species fails to capture trends in abundance or activity, and also therefore the trends in expected value or loss of the ecosystem service or disservice (Baker et al., 2019; Callaghan et al., 2024).

From an ecosystem resilience perspective, trends in population size can highlight a number of ecological dynamics and act as early warning systems of demographic stress. Smaller populations are more vulnerable to shocks, as the smaller the population, the more important each member is to the survival of the group (Kaye et al., 2019). Even normal ecological processes like predation or disease can have a disproportionately high impact on a small population, while severe ecological disturbances such as natural disasters can be lethal. Extinction debt—where a species faces local extinction because the population has fallen below the threshold needed to sustain itself—can take years or decades to pay (Liao et al., 2022), with populations initially showing only a gradual decline that accelerates over time (Fagan & Holmes, 2006). Similarly, isolated patches of habitat, even those with a large population of a particular species, can function as population sinks dependent on immigration from other patches to sustain themselves. The key driver of this dynamic is a population with a reproductive rate lower than the rate of mortality. In these cases, if the connectivity to the larger patch were to disappear, local extinction would ensue (Pulliam, 1988).

In the last two cases, even with accurate measures of abundance, it can be tricky to capture the long‐term viability of populations. Without such measures, however, the few warning signs of an impending collapse will be missed entirely. As with all indicators, those incorporating abundance must do so with the ecology of the focal species in mind. Abundance measures can fluctuate by orders of magnitude due to natural demographic and environmental cycles, which, if unaccounted for, can lead to serious misinterpretation (Santini et al., 2017).

## TIMELINES FOR ESTABLISHING BIOLOGICAL TRENDS

At the surface level, to “monitor” a species means simply to track the change in its behavior, physical condition, distribution, or abundance over time. With such data, we seek to understand the patterns of individual and population dynamics, notice when they change, and explain why. The key word here is “change,” because to evaluate whether something about a species has changed in a way that indicates a change in status or that has implications for ecological dynamics, we must first know what is “normal.”

Human activity has affected all habitats on Earth to some degree. While the rate and magnitude of this impact have increased in recent years, humanity's influence on the natural world began thousands of years ago. The ecosystems we observe and interact with today are therefore neither pristine, that is, absent human influence, nor simply degraded; they are the products of complex, long‐term interactions between human communities and ecological processes, some complementary, some antagonistic. Establishing what is “normal” is therefore rarely straightforward.

One recurring challenge in monitoring change is the use of a single year as the fixed baseline against which subsequent observations are evaluated, a practice common to many offset and credit schemes (Wunder et al., 2025; zu Ermgassen et al., 2021). The UK's Biodiversity Net Gain framework, for example, quantifies change against a baseline established in a single survey year, even if the monitoring is expected to continue for decades. The concern is not that an ecologist would attempt to infer a trend from a single temporal snapshot, but that the reference data are a noisy point estimate rather than an established trend, which risks misrepresenting ecological patterns (Claus et al., 2026; Knape, 2023). On a short timeline, patterns in the abundance of a species will not have been defined, and outliers caused by unusual climatic or ecological events, human disturbance, or changing migration patterns will not be recognized for what they are (White, 2019). Data limitations, including the lack of longitudinal data and data quality issues introduced by sampling errors, seasonal change, day‐to‐day variation, and uncertainty during measurement, will further influence how the data are interpreted.

A related problem is the timeline along which change is measured after an intervention intended to improve biodiversity. Species react to environmental change at different rates, governed by life history traits such as dispersal ability, reproductive rate, and longevity (Pedley & Dolman, 2014). Generalist species that are tolerant of disturbance and exhibit a fast reproductive rate and large dispersal ability will be quick to colonize open niches or exploit changes in connectivity, while habitat specialists with a low dispersal ability will not (Summerville et al., 2006). The intervals at which data are collected post‐intervention will therefore capture different taxa at different points in their response trajectories.

A separate issue has to do with where baselines are set. Ecological baselines are routinely defined from the earliest available data, and recovery targets set against pre‐disturbance conditions that were already impoverished (Mihoub et al., 2017; Rodrigues et al., 2019). For many monitoring schemes, this is entirely appropriate. The aim is not to return to a pre‐disturbance ideal, but rather to see net improvement relative to current conditions. However, the significance of any improvement is only relevant in the context of the baseline, and when the baseline is established from an already‐impoverished state, then this state becomes the “normal.” As every new project and generation reestablishes the baseline, the conditions that define normal slide further downward, dragging with them what counts as “improved,” a phenomenon known as the shifting baseline syndrome (Pauly, 1995; Soga & Gaston, 2018). At the landscape or regional scale, many independent projects can each report a net gain while the cumulative trajectory points downward, a trend obscured by the ever‐lowering benchmark against which success is measured.

More broadly, these considerations point to a fundamental distinction between static and dynamic approaches to biodiversity monitoring. Static approaches—those that compare a snapshot of biodiversity against a fixed baseline—are inherently limited in their ability to capture the trajectory and direction of ecological change, let alone the processes driving it. Dynamic approaches, by contrast, treat biodiversity as an ongoing, multi‐scalar process shaped by ecological, climatic, and human forces, and seek to characterize the rate, direction, and drivers of change over time (Perfecto et al., 2009).

The need for long‐term data is well known to ecologists; however, practical and financial constraints often limit study timelines, particularly in agroecological contexts (Gent et al., 2026; Kleijn & Sutherland, 2003; Rau et al., 2020; Redhead et al., 2022; West et al., 2023). A second‐order meta‐analysis that examined the effect of long‐term agricultural diversification on, among other things, biodiversity found that 78% of the 211 studies that focused on aboveground biodiversity ran for less than 10 years, while 55% ran for less than 5 years (Raveloaritiana & Wanger, 2026). To accurately capture the impact of a new practice or conservation effort, change must be viewed in the long‐term and against trends quantified over statistically significant and taxon‐specific timelines (Knape, 2023). Pairing quantitative data with testimony from local experts has been shown to reduce these uncertainties (Beaudreau & Levin, 2014).

## SPATIAL SCALE

There is no single appropriate spatial scale best suited for evaluating the environmental sustainability of agricultural practices (Dale et al., 2013). An individual farm can range in size from less than an acre to thousands of acres, while the effect of any given farming practice, both beneficial and harmful, can be contained within a few meters, or be felt kilometers away from its source (Albaseer et al., 2025). Some impacts are also emergent: Their landscape‐scale magnitude exceeds the summed contribution of individual farms because the effects of each farm compound on one another. Habitat connectivity is a prime example. No amount of conservation on a single farm can create connectivity on its own, but even small strips of natural habitat, once linked across farms, create a connected landscape (Tscharntke et al., 2005).

Likewise, different species are affected by and respond to changes in their environment at different scales, interacting with similar scale‐dependent patterns in natural processes, ecosystem functions, and community dynamics (Mitchell et al., 2024; Pease, 2024; Wiens, 1989). Most macroorganisms rely on habitats and resources outside of any one farm and respond to changes well beyond a farm's boundaries, whether this be the composition, connectivity, or quality of the surrounding habitats, or environmental stresses introduced by a neighboring farm (Gabriel et al., 2010; Tscharntke et al., 2005).

The indicators and methods with which we choose to monitor biodiversity can perform very differently depending on the scale of interest. Indeed, bioindicator species, which are often chosen to track the environmental impact of agricultural practices, can show different and even contrasting responses at different life stages when monitored at different scales (McMahon et al., 2012). An effective monitoring methodology is one that is tailored to the spatial scale of the ecological response, rather than to the spatial impact of the farming practice.

A practice implemented at the field level, such as the installation of flowering strips, is spatially bounded and easily delineated. Even so, the scale at which organisms respond to such a practice depends heavily on the ecology of the species in question. Many ground beetles, for example, have limited dispersal abilities, and their presence and abundance are tightly linked to the local habitat conditions, making them well‐suited proxies for detecting the impact of a flowering strip within a few hundred meters (Rainio & Niemelä, 2003). The same taxa also contain highly mobile generalist species, which, like many species of solitary bee that are also commonly used to investigate the effect of local agricultural conditions, depend not only on local floral resources but also on the availability and accessibility of nesting habitat, factors that may be absent in the immediate vicinity of the flowering strip. Their response, or lack thereof, can only be meaningfully interpreted at spatial scales on the order of many hundreds to thousands of meters (Galpern et al., 2021; Steffan‐Dewenter et al., 2002).

For detecting the cumulative effect of agricultural practices across a region, however, the principle is reversed and favors the use of communities structured primarily by landscape‐level processes. Farmland bird communities, for example, navigate conditions across large home ranges and respond to landscape composition and environmental quality at broad spatial extents, requiring coarse‐grained monitoring over large areas to detect meaningful trends (Gregory et al., 2005). Fine‐grained surveys that are concentrated in a few areas might suggest local recovery while missing regional decline, or vice versa. A standard route of balancing the grain and extent is to use multiple fine‐grained surveys which, when spatially representative enough, can detect change at both local and regional scales (Wood et al., 2017). Another strategy is to use species that are effective indicators at small and large scales, tracking overall landscape quality at the regional level as well as showing strong, predictable responses to individual landscape elements at the local level (Padoa‐Schioppa et al., 2006).

The biodiversity value of a farm is not solely a property of the farm itself, but emerges from its interaction with the surrounding landscape, such as the ecological connectivity it provides or obstructs, the regulating and supporting ecosystem services it provides, and the management‐driven ecological and co‐evolutionary dynamics it supports or suppresses (Bennett et al., 2021; Nabhan, 2012). It is imperative, therefore, that biodiversity monitoring, whether to track the state of ecosystem health or the impact of agricultural practices on it, be conducted at a spatial scale appropriate for the objective and context at hand. Tools and techniques for monitoring biodiversity affect the scales and resolutions at which data can be collected, but more important is knowledge of the manner and extent of impact by a particular practice, and the ecological response of the species or communities being monitored.

## BIODIVERSITY FOR WHAT?

An ecosystem is more than the sum of its parts. Outwardly lush ecosystems can be experiencing mass local extinction (Redford, 1992), while seemingly sparse ecosystems can exhibit high primary productivity (Ahlström et al., 2015) and high resilience (Hoover et al., 2014; Xu et al., 2021). The image of a “healthy ecosystem” is most often represented by an untouched tropical forest. And, as expressed by the GBF's 30 × 30 goal (Convention on Biological Diversity, 2022), protecting “pristine” natural habitats is indeed an essential part of conserving global biodiversity. But natural habitats are only one of the priorities outlined by the GBF for protecting biodiversity. The GBF also speaks of ecological functions—both for ecosystem resilience and to meet human needs. It speaks of habitat area, connectivity, and quality. It speaks of biodiversity from the scale of globally significant ecosystems to individual species, noting the importance of protecting those going extinct and managing those that are invasive. It speaks of genetic diversity of both wild and domestic species, and the implications for adaptive capacity should their current decline continue. And finally, it speaks of biodiversity in agricultural and urban landscapes, because, while untouched habitats might be hotspots for biodiversity, most of the world is not untouched, and human‐modified landscapes, agriculture foremost among them, are an extremely important battleground in the fight to protect nature.

The sheer variety of ecological features outlined as priorities in international frameworks such as the GBF highlights the complexity of the natural world, the myriad interdependent processes and functions, working at different scales and timelines, that sustain natural ecosystems. Our relationship with the natural world is just as intricate. Human society relies on and damages natural ecosystems in just as many ways and on just as many scales and timelines. Following the priorities for conservation, the GBF also highlights specific human impacts that must be minimized to protect biodiversity. These range from species‐level threats due to poaching and human–wildlife conflict to global threats due to pollution and excessive resource extraction.

Adding to this complexity are features such as ecological health and resilience, both system‐level emergent properties, which depend on key ecological functions and dynamics that enable the ecosystem to thrive in the face of disturbance (Beller et al., 2019; Oliver et al., 2015). These emergent properties are not created by any one species or taxa, but result from the cumulative interaction of the system's many parts. Which species, and how many of each, matter. But features like resilience are emergent phenomena that are formed in the aggregate of interacting structures and dynamics at different scales. If healthy ecosystems are the end goal, then comparing the change in a species, taxa, or habitat quality between two systems can be misleading. To a certain degree, bioindicator species sensitive to environmental conditions can be used to make inferences about the broader ecosystem (Carignan & Villard, 2002), but in general, first‐order indicators when considered in isolation do not speak to the question at hand, and therefore cannot effectively capture ecosystem‐level emergent properties such as resilience (Hillebrand et al., 2018; Oliver et al., 2015). Multiple complementary metrics or indicators tracked over time can partially bridge this gap and provide approximations of system‐level properties.

The resources for a conservation effort can be spent in multiple ways. Protecting a specific species might be best served by targeting habitat features necessary for that species (Johnson & Ray, 2025). Increasing the total number of species, regardless of their identity or function, might be best served by expanding the total amount of natural habitat (Fahrig, 2003) and connecting new patches to a “source” patch from which species can immigrate (Knop et al., 2011; Newmark et al., 2017). Increasing the provisioning of an ecosystem service, for example pollination or pest control, might involve the construction of flowering strips adjacent to crops (Albrecht et al., 2020). Meanwhile, the construction of habitat islands or linear elements in an otherwise impermeable agricultural matrix would increase the connectivity and permeability of the matrix. Such a placement might also greatly improve the resilience of the ecosystem as landscapes with intermediate levels of connectivity and habitat amounts are both highly susceptible to local extinction and also show the greatest potential for recovery following conservation interventions (Tambosi et al., 2014).

Which raises the question, “what are we protecting biodiversity for?” If the goal is to protect a specific rare species, then monitoring the population of that one species might be sufficient to track progress. However, when the goal is more holistic, as many argue should be the case in most agroecosystems, such as supporting adaptation to climate change among threatened species, progress should be measured as change in ecosystem health and condition relative to these goals (Bennett, 2017). If the end result of a project is a sparse but stable ecosystem, but the project began when the ecosystem was severely degraded, then this outcome, by many indicators, would be considered a success. The same would not be true if an already stable ecosystem continued on its trajectory toward a pristine ideal. Of the two scenarios, only the former would meet the criteria for additionality. This being the case, policy, regulation, and incentives should aim to capture and reward holistic improvements in ecosystem health, or else risk creating perverse incentives for projects and indicators that show the greatest numerical gain in the shortest time, regardless of the local ecological and sociological needs (Aide, 2024; Marshall et al., 2020).

## METHODS AND TECHNOLOGIES FOR BIODIVERSITY MONITORING

A plethora of tools and methods now exist designed to detect, measure, track, and analyze the natural world (August et al., 2015; van Klink et al., 2024). Each of these methods captures a different facet of biodiversity, from presence–absence data of target species to species density of select taxa, and generalized indices of ecosystem complexity (a non‐exhaustive list is detailed in Table 2). Each method also comes with its own limitations and biases that must be acknowledged and accounted for. For example, there is a global North–South bias in many types of classifiers that are trained on data primarily sourced from the global North, making them more accurate/effective in these regions (Jeantet & Dufourq, 2023; Sánchez Herrera et al., 2024).

**TABLE 2:** A non‐exhaustive list of common biodiversity monitoring tools and methods, and key considerations of the data they collect.

Approach	Indicator	Considerations
Manual and passive acoustic monitoring	Soundscapes (Darras et al., 2025), and the presence, abundance, behavior, and density of multiple faunal taxa, primarily birds (Pérez‐Granados & Traba, 2021), bats (Sugai et al., 2019), amphibians (De Araújo et al., 2024), and insects (Faiß & Stowell, 2023; Rodríguez Ballesteros et al., 2024).	Manual methods require oftentimes extended periods of expert fieldwork. Passive methods are less demanding on field, but then can require expert input during analysis, the extent of which is determined by the quality of the acoustic classifier, which are often geographically or taxonomically biased (Gibb et al., 2019) and vulnerable to domain shifts (Navine et al., 2024). Techniques for abundance estimation from acoustic data, meanwhile, remain methodologically challenging and species‐dependent (Fiss et al., 2024).
Camera traps	Identification of flora and many faunal taxa, including reptiles, insects, birds, bats, and mammals, to the genus, species, and individual level (Bjerge et al., 2022; Burton et al., 2015; Delisle et al., 2021), from which information on activity and density can be derived (Schneider et al., 2022; Vélez et al., 2023).	Manual identification of animals in images and videos can be time‐consuming. Computer vision models show varying accuracy with taxa and environment, and are vulnerable to domain shifts and imbalances in training data (Schneider et al., 2020).
Environmental DNA (eDNA)	Species identity and richness of multiple aboveground taxa, including rare, invasive, and traditionally hard to detect species (Kestel et al., 2022; Macher et al., 2023). In some cases, this method can also reveal details of a species' functional niche (Wirta et al., 2022). Species identity with relative abundance can be captured in the case of soil microbiota (Gellie et al., 2017).	In most cases, eDNA cannot be used to estimate abundance (Elbrecht & Leese, 2015; Saccò et al., 2022). While relative abundance can be estimated in the case of soil microbiota, niche expert input and detailed physiological information on soil microorganisms is often needed to accurately contextualize the results (Wilhelm et al., 2023). Analyzing environmental samples is expensive and requires labs with specialized infrastructure. Further, accurately assigning a DNA sequence to an established taxonomic group can be a computational and statistical challenge (Ruppert et al., 2019).
Traps and nets	Species ID of insects, birds, bats, and some small mammals (Appel et al., 2021; Blake et al., 2023; Freeman et al., 2022; McCravy, 2018). In some cases, relative abundance (Wiewel et al., 2009) and population estimates (Hammond & Anthony, 2006) can also be derived.	Mist nets and harp traps require highly trained individuals to set up and man for the entire period of deployment. They are therefore difficult to deploy at scale. Insect traps require less manual effort but vary in their cost‐effectiveness (Hoffmann & Stoll, 2025).
Airborne sensors	Species richness and diversity of many plant species. Species‐level identification and abundance estimates of some animal species (Descamps et al., 2011; Groom et al., 2011) and various measures of floral biodiversity and habitat quality (Petrou et al., 2015).	The use of drones can be expensive, require significant technical expertise, have limited flight time, and in many contexts be subject to restrictive regulation (Crutsinger et al., 2016).
Satellite‐based remote sensing	Habitat features include connectivity, fragmentation, various indicators of habitat structure and productivity (O'Connor et al., 2015; Reddy, 2021). In some cases, species presence, richness, and distribution can be indirectly estimated through known links between species behavior and habitat characteristics (Leyequien et al., 2007).	Despite ever‐increasing spatial resolution, satellite‐based analyses are still limited in the types of data they can effectively capture. Direct monitoring of individual animals is only possible in a very few cases—such as elephants (Duporge et al., 2021) and elephant seals (McMahon et al., 2014).
Indigenous guardians, rangers, sentinels, citizen science, and other local stewardship	Indigenous‐led guardianship engages with multiple kinds of observational and historical data to ensure ecosystem health and resilience (Brondízio et al., 2021). These data can include species presence/absence and abundance, based on historical baselines, informed by oral histories or other local datasets, distribution, changes in distribution, phenology and changes in phenology, plant/animal condition of harvested species (Huntington, 2000).	Stewards working with a depth of ecological knowledge, often held and shared orally, bring highly contextual expertise; cross‐cultural sharing requires trust and an ethical space in which people working with different forms of expertise and data can share and learn from one another (Bartlett et al., 2012; Moola & Roth, 2019). Data quality can vary based on the experience of the collector.

As already noted, different indicators of biodiversity involve different degrees of conceptual complexity—some are relatively straightforward, such as species richness, while others are much more contextual and layered, such as cultural keystone species (Garibaldi & Turner, 2004). In general, the most directly observable indicators are the easiest to measure and monitor, but also the weakest in terms of the inferences about biodiversity they can support on their own (Gotelli & Colwell, 2001). For example, common field biology methods for measuring biodiversity such as camera traps, acoustic recorders, and pitfall traps are relatively easy to deploy, but their interpretation, especially for complex biodiversity principles like ecosystem health, requires numerous assumptions or other supporting information (Burton et al., 2015; Prado et al., 2017). Conversely, Indigenous and local knowledge about keystone species abundance is more subjective in nature and involves more complex, collaborative social science methods to measure and monitor, but their holism and integrative nature can support stronger inferences about different biodiversity principles, particularly when complemented by Western scientific approaches (Berkes & Berkes, 2009; Ijatuyi et al., 2025).

The lack of high‐quality, verifiable data, and the difficulty of attributing change to a conservation intervention, is often pointed to as a limiting factor for investment in projects that protect biodiversity (CPIC, 2021; zu Ermgassen et al., 2025). However, in a system where low profit margins are the norm, which is generally true at the farm level, the lack of investment can limit access to the very technologies needed to collect the sought‐after data. When hard‐to‐collect data collected through expensive or inaccessible methods are made must‐haves in monitoring frameworks, the beneficiaries of said framework will be limited to demographics that already have access to funds and/or expertise. Expanding efforts to monitor and protect biodiversity relies on promoting tools that are accessible and affordable. This is important to remember when championing a method or technology and pushing for its incorporation into policy or incentive structures.

## DATA POLITICS AND OWNERSHIP IN THE AGE OF BIG DATA

In addition to the question above about what biodiversity is important to protect, a corollary question exists: “Why and how are we monitoring biodiversity?” It would be naive to assume the simplest answer—in order to protect it; all data are political, reflections of the societal values and agendas that motivate their collection (Goldman et al., 2018). A thorough consideration of how we measure and monitor biodiversity, therefore, calls for a proactive exploration of who is and is not privileged by the definitions, frameworks, and architecture that are codified in policy or markets (Loring et al., 2021). For example, currently, private sector‐led innovation is widespread in the biodiversity data collection space, with a focus on techniques that produce reportable and verifiable data that serve the needs of supply chain actors (Beck‐O'Brien & Bringezu, 2021; Gonzalez et al., 2026), ostensibly under the auspices of providing evidence that their products are climate or nature friendly (Allen et al., 2024).

These data are either collected via state and non‐state technologies such as satellites, or alternatively collected on private farmland; either way they are often collected in service of large‐scale food systems actors interested in financializing this information. Tools exist today that can be used to monitor everything from forest restoration efforts to cropping practices and field conditions remotely, and at high resolutions, without the knowledge or consent of landowners or other territorial rightsholders. If data for reporting on or commodifying biodiversity have value, then the tools to collect these data are a gateway to said value. These data, therefore, have the power to add to or detract from the agency of people in the lands and ecosystems they purportedly represent. Who owns these data is not necessarily clear‐cut, as laws and precedents related to intellectual property and corporate privacy could give agribusinesses much power over who can and cannot access data collected at their behest (Fraser, 2019).

Consent is a related matter; it takes conscious effort on the part of practitioners to ensure that all relevant parties are fully aware of what data are being collected and agree to the protocols for dealing with privacy and security of these data. Some of the tools listed above, including drones, acoustic recorders, and camera traps, are capable of capturing private and sensitive data unrelated to the study at hand. At the local or even subregional scale, there may well exist expectations that a farm is only included in a study if the farmer provides free and informed consent. However, it would be impractical to expect a country‐level remote‐sensed study of farmland extent, for example, to be required to get opt‐in consent of every farmer in a country. In between these two scales, things become trickier.

Global environmental problems are no doubt progressing at such a rate that we must utilize every tool at our disposal to ensure that our understanding keeps pace with the change. Many of the latest tools and big datasets are attractive options for quickly identifying scalable solutions to pressing problems (Hampton et al., 2013). That being said, every person, community, and culture has the right to privacy, agency, and self‐determination. Data about biodiversity will invariably be used to inform decisions and priorities that impact people's lives directly and indirectly, but data cannot substitute for the will of the people living in the regions that these data represent. And, unlike many North American Indigenous societies, which have processes for consent at the level of the community and Tribal Nation, Western governments, corporations, and scientists generally approach large‐scale data collection and representation as not involving consent at all (Wyborn & Evans, 2021). Whether and how our approaches to compiling, managing, and acting on large biodiversity datasets address the need to protect data access and sovereignty will be a reflection of whether and how our societies respect these basic rights in general.

## BALANCING PRODUCTIVITY, PROFITS, CARBON, AND NATURE

Farmers manage almost half of the habitable area of the world. Despite the best efforts of some, global food systems collectively have an enormous net‐negative impact on biodiversity, environmental quality, and greenhouse gas emissions. There is no doubt that global agriculture, and the food systems around it, is responsible for a significant fraction of humans' impact on nature. And yet to lay this responsibility squarely on the shoulders of farmers would be a dangerous misunderstanding of the food system, one that could blind us to the levers of change necessary to make agriculture more sustainable.

The demand for ever‐increasing agricultural productivity is not an a priori fact of food systems, but a political condition shaped by over a century of corporate consolidation across the agri‐food chain (Clapp, 2023). A small number of firms now exert enormous influence over global seed, agrochemical, grain trading, and retail markets, creating structural pressures that lock farmers into high‐input, high‐output production models regardless of their individual preferences or local ecological conditions (Clapp, 2021; Clapp & Fuchs, 2009). These dynamics are inseparable from broader processes of accumulation by dispossession (Harvey, 2012), whereby land and resources are concentrated through mechanisms ranging from colonial enclosure to contemporary large‐scale land acquisitions—often characterized as “land grabs”—that displace smallholders and Indigenous communities in favor of industrial commodity production (Borras & Franco, 2012; McMichael, 2013). Productivity, in this light, is less a value‐neutral measure of agricultural system performance than an outcome of a political and economic system that prioritizes output and return at the expense of all other social and environmental goals.

A farmer sits in the center of a complicated web of goals, restrictions, values, and incentives that vary extensively from place to place around the world. Farmers invariably must reconcile personal and communal needs and values with short‐ and long‐term profitability in a landscape of increasingly unpredictable weather (Eitzinger et al., 2018), complex and sometimes perverse regulatory and insurance structures (Möhring, Dalhaus, et al., 2020; Müller et al., 2017), historical agreements with suppliers (Mase & Prokopy, 2014), and an agroecosystem struggling to stay productive. Despite the potential of ecologically intensive practices to shift food production—such as through agroecological cropping practices, or setting aside marginal land for natural habitats—a motivated and well‐informed farmer would still struggle to change their practices in a climate that first and foremost rewards agricultural productivity. This climate is not accidental; it is maintained by a consolidated agribusiness sector that maintains overwhelming control of input supply, commodity markets, and retail, narrowing the viable options available to farmers and entrenching farmers in intensive production (Clapp, 2023). While opportunities such as biodiversity and carbon markets are emerging that compensate farmers for more environmentally conscious management practices, the barriers to access these opportunities remain high.

In addition to protecting biodiversity, agriculture is under increasing scrutiny for its impacts on soil erosion, pesticide and fertilizer pollution, and GHG emissions, as well as for their potential value as carbon sinks via sequestration in the soil (Lal, 2004; Mielcarek‐Bocheńska & Rzeźnik, 2021; Möhring, Ingold, et al., 2020). The reduction of pollution and emissions is no less important than the protection of biodiversity, and in a broader sense, they are all part of the same ultimate goal to minimize our impact on the world. To some farmers, biodiversity joins a long list of incentive regulation structures that attempt to define how they manage their land. To others, particularly financially insecure smallholders with little flexibility, biodiversity and carbon simply do not feature in their day‐to‐day decision‐making unless they can be tied to immediate profits.

This is a system riddled with contradictions and trade‐offs (Rodríguez et al., 2006). Practices that improve the sustainability of a farm in one dimension can reduce it in another. Trade‐offs are rife and often seen between provisioning services and regulatory and cultural services (Raudsepp‐Hearne et al., 2010). Even more tenuous are decisions that weigh small impacts on a large scale against large impacts on a local scale. There is no “ideal balance” that addresses every concern in an equitable way; rather there is an evolving, subjective matrix of concerns that can vary from farm to farm. Practitioners coming into this conversation with a certain worldview, expertise, and mandate should be aware that local priorities, cultures, and contexts might have a very different view of what is important. Which leads us to the next point…

## BIODIVERSITY FOR WHOM?

Wyborn et al. posed the question, “What is a desirable biodiversity future, and for who?” (Wyborn et al., 2021). From global organizations to individual farmers, there is an enormous variety and diversity of actors and institutions involved in the biodiversity space, each aware of, interested in, or worried about a different facet or function of natural ecosystems. Compounding this intricacy is the fact that not protecting nature is a costly proposition, one where the costs extend, directly or indirectly, to all aspects of life, from food security and health to culture and happiness. Biodiversity is therefore inherently political. Every decision made to protect biodiversity, including the structure of incentive frameworks, the overarching goals of a project, and the indicators and scales of measurement, all ascribe a value to biodiversity that prioritizes some aspects of it over others. And, as the value of biodiversity is so tightly linked to other dimensions of life, decisions to protect biodiversity also implicitly ascribe value to these other dimensions. Below we outline some of the actors and arenas of decision‐making in this space.

### The global arena

At the global scale, overarching priorities for protecting nature are created by intergovernmental panels and international collaborations. Some of these, such as the Sustainable Development Goals, set biodiversity alongside and at par with other priorities such as food security and justice. Other frameworks, such as the GBF, focus primarily on biodiversity, the threats facing it, and the value it provides. Non‐biodiversity aspects such as agriculture come in only in the context of the impacts they have on biodiversity and the value they derive from it. These bodies seek to bring about change at the global scale, with a view to avert threats to humanity. They do not, and indeed cannot, incorporate regional contexts, or measure the trade‐offs at the local scale, but they can through multilateral agreements establish the frameworks for action and accountability by which regional and country‐level action must happen. Nationally defined contributions (NDCs) to action on climate change and National Biodiversity Strategies and Action Plans (NBSAPs) are two important examples.

### The national arena

At the national level, international frameworks butt heads with other national priorities, financial realities, and various flavors of politicking. The costs and benefits of investing in biodiversity are weighed against what are, or are perceived to be, more important, politically valuable, or practically feasible matters such as food security, economic growth, health, and defense. National priorities, which often experience regular reshuffling, are also influenced by a country's trajectory and historical path dependencies. These trajectories may be shaped by resource dependencies, colonial legacies, and past economic models, all of which affect how biodiversity is valued and integrated into policy decisions.

### Private industry

The growing interest in biodiversity assets swells the role of private enterprise in protecting nature. A private company could be motivated to invest in monitoring or improving biodiversity for a number of reasons, including the need to report on their impacts on biodiversity, to minimize risk within supply chains dependent on biodiversity, and to improve their image in the public's eye. Each motivation shapes their approach to biodiversity differently and influences how and where they choose to invest in conservation, and whom they choose to engage with along the way.

Financial considerations will no doubt exert a strong influence on how private companies choose to approach the issue of biodiversity. This carries several risks, including creating perverse incentives for quick wins, prioritizing indicators likely to show rapid gains, choosing overly simplistic, reductive, or disconnected methods that misrepresent ecological realities, or adopting standardized, context‐independent approaches that ignore local needs and priorities in the quest to hit a target. There have also been concerns raised about the limitations of market‐led protection of nature. Most ecosystem services being non‐rival and/or non‐excludable—that is, common goods that cannot be restricted or whose value to one party is not diminished through use by another (Costanza et al., 2014)—there is a worry that if left to conventional market forces, natural systems will be inappropriately valued and therefore poorly protected (Costanza, 2006). Likewise, market commodities are often considered substitutable—corn from one locale can be replaced with corn from another with negligible instrumental loss—but ecosystems are not substitutable—you cannot replace a wetland in one locale with a wetland in a second without creating tangible losses of both inherent and instrumental value (Ang & Van Passel, 2012). Nevertheless, we concede that the momentum behind neoliberal solutions in private industry and governments is likely too great to overcome altogether on a reasonable timeline. And, given that it is clear that industry as a whole is becoming a large presence in biodiversity conversations (Panwar et al., 2023), we believe that efforts must now be made to ensure both that ecosystem valuations are as holistic as possible and that noncommercial forces are empowered to complement the priorities of private actors.

### Indigenous perspective

Indigenous communities around the world are well known for their track record of protecting and enhancing biodiversity through their livelihood and stewardship practices. Indeed in many parts of the world, the “natural baseline” that biodiversity proponents seek to protect was in fact at least partially shaped by the intents and actions of Indigenous Peoples. Numerous studies demonstrate that Indigenous Peoples have been, and continue to be, critical to safeguarding biodiversity around the world (Garnett et al., 2018). While there is no doubt that biodiversity provides numerous services that are essential to Indigenous Peoples' continuance of their traditional lifeways and cultures, their vested interest extends well beyond this instrumental dimension; many Indigenous communities around the world consider nonhuman species to be kin and biodiversity stewardship within their traditional territories to be a birthright responsibility.

Historically, Indigenous agroecosystems and wildlife and land management systems around the world promoted biodiversity in addition to provisioning food and other material and cultural resources (Agnoletti & Santoro, 2022; Lepofsky et al., 2021). As noted, however, today the conversation around biodiversity monitoring and protection in agroecosystems is largely shaped around the issues and priorities facing large‐scale industrial food supply chains, even if the lands in question that are owned and farmed remain unceded Indigenous territories. These bring critical environmental justice issues into the discussion—As we develop new ways to conceptualize, quantify, and compensate people for actions that result in ecological outcomes, we have to see these new systems in light of the broader and still harmful colonial systems that our reimagined approaches to biodiversity are perpetuating.

### The local arena

Biodiversity is only one factor in the livelihood‐sustaining decisions facing farmers, who have their own set of needs, values, and ideas for their land, which range from practical and financial to aesthetic and sentimental, all of which influence their style of management. National and regional policies can run counter to these factors, and even if perfectly aligned, can face any number of barriers to implementation. This can include a farmer's access to and trust in information regarding the current science, tools, and policy for sustainable farming (Solano et al., 2003; Vecchio et al., 2020); access to seed, technology, markets, and infrastructure; path dependencies related to equipment, artificial inputs, and insurance (Hackfort, 2023; Spangler et al., 2022); and individual values and dispositions toward risk and change, often influenced by socio‐demographics (Edwards‐Jones, 2006; Hayden et al., 2021).

The decisions made by a farmer may not align with what experts in various fields judge to be best, but “best” is a subjective and fluid concept. It changes with evolving priorities, timelines, and perspectives. Expert‐informed top down policies are often aimed at improvement in the average and can therefore overlook the individual. Yet, at the end of the day, it is the individual whose decisions dictate the future of their farm and all the implications contained therein.

### Summary

The aim of this section is not to compare or judge the merits of any one actor, but rather to highlight the potential for conflict when multiple actors and value systems come together. Whether or not we are aware of it, every decision made in this space assumes a philosophy toward biodiversity protection. Consciously or unconsciously, this philosophy will impose itself on different communities, ecosystems, and locations by selectively providing the benefits to some over others.

Who receives these benefits and who does not often boils down to a question of access. Access to markets where biodiversity assets are traded, to supply chains where sustainable farming produce is valued, to incentives for ecologically conscious practices, to information, skills, and technical resources necessary to collect and analyze data, and to conversations where decisions about priorities, responsibilities, and stewardship are made. Ostensibly, the overarching goal is to protect nature, and yet nature itself does not have a voice. Instead, we hope that nature is represented in the aggregate of all the voices in the conversation. However, not all voices in this conversation carry equal weight, and when different sets of values and priorities clash, it is inevitable that some will override others. By default, the weight of power tends to rest with a few actors—those with money, influence over policy, and access of various sorts. It is often the case that equity is only reached when power, whatever its manifestation, is consciously and deliberately shared in the hope that it brings the consensus closer to the “right” decision.

There are no perfectly “right” decisions, but there are decisions that create undeniable harms to the biosphere. And in a food system that supports such a large fraction of humanity, getting it wrong has serious and far‐reaching consequences for people and nature. To avoid this, practitioners must adopt an inclusive approach that considers the concerns of everyone at the table, having first ensured that everyone *is* at the table.

## CONCLUSION

By the end of this paper, a practitioner new to the agrobiodiversity space might have thrown their hands up in exasperation. The considerations we lay out, if taken seriously, increase, rather than reduce the complexity of monitoring biodiversity (whichever aspect over whatever timeline) in agricultural settings. The simple fact of the matter is that there are no easy answers, and even the solutions that do exist are often lost in the deluge of information being constantly generated. As Heller and Zavaleta pointed out, practitioners in the agricultural space need tools to guide them through the “sea of adaptation ideas” (Heller & Zavaleta, 2009), while accounting for local priorities, social‐ecological contexts, and financial, technical, and logistical constraints. Part of this will involve simplifying the regulatory and insurance landscape within which those in the agricultural and agroecological space operate. To sustain both the health of ecosystems and the contributions those ecosystems make to people and society, we need to move the conversation beyond “silver bullet” solutions. In the past, the single‐minded focus has been on the scale and efficiency of production; today, we see a similar single‐mindedness emerging around so‐called “carbon tunnel vision” (Deivanayagam & Osborne, 2023). Without clear direction, the challenge of protecting nature risks falling into the same trap of reductive indicators informing ineffective solutions. Nonetheless, progress does have to be measured. To do so effectively, we must choose approaches, methods, and tools that appropriately collect and situate biodiversity data within the ecological and sociological landscape they came from. This will require acknowledging the inherently contextual nature of biodiversity, not just how indicators speak to ecological state, but how nature—species, communities, functions, and processes—interacts with, is affected by, and impacts the wider world.

Different communities of practice will find different entry points into this complexity. For policymakers designing biodiversity incentive frameworks, the central message is that indicators and baselines must be ecologically grounded, context‐sensitive, and resistant to the perverse incentives that emerge when ecological outcomes are reduced to simple numerical targets. For private sector actors, it is essential to recognize that ecosystems are not substitutable commodities and that market‐led approaches must be complemented by noncommercial mechanisms that protect ecological and social values the market cannot price. For data scientists developing monitoring tools, the imperative is to ensure that tools are accessible and that data sovereignty is respected. And for all practitioners, the work of Indigenous communities, agroecologists, food sovereignty movements, and community‐based conservation programs offers not just inspiration but tested models that should be centered rather than peripheralized in the design of new systems. Many of the ideas in this paper are not new, but well established in certain disciplines. The challenge is ensuring they are heard and taken seriously wherever consequential decisions about biodiversity's future are being made.

Importantly, questions of land tenure, sovereignty, and democratic control over land use decisions are also fundamental to determining whether biodiversity gains are equitable and durable, but lie beyond the scope of the present review. We direct readers to the growing literature on food sovereignty, peasant movements, and agrarian justice for essential context (Altieri & Toledo, 2011; Gonzalez, 2021; Perfecto et al., 2009).

On its current trajectory, the expansion and intensification of agriculture is likely to continue causing severe damage to the Earth's biosphere. Much of the knowledge and tools needed to limit that damage already exist, but implementing change in such a complex system on a relevant timeline is an enormous challenge. As ecological concepts previously confined to academia now enter board rooms and investment portfolios, it is ever more important that biodiversity in agriculture not be shoehorned into existing structures for carbon assets or conservation projects, or be reduced to overly simplified, a‐cultural and a‐political measures of ecosystem condition. Complicated though it is, biodiversity is only one element in a very complex global food system that must balance the environmental impacts of agriculture against local, national, and global priorities for food security, livelihoods, and environmental security writ large (Rockström et al., 2009). The greatest, most equitable impact will come when there is buy‐in and collaboration from all the actors in this space, and a willingness to embrace the complexity so created.

## AUTHOR CONTRIBUTIONS

*Conceptualization*: Iqbal Singh Bhalla. *Writing—original draft*: Iqbal Singh Bhalla and Philip A. Loring. *Writing—review and editing*: Iqbal Singh Bhalla and Philip A. Loring. *Supervision*: Philip A. Loring.

## CONFLICT OF INTEREST STATEMENT

The authors declare no conflicts of interest.
